# The model marine diatom *Thalassiosira pseudonana *likely descended from a freshwater ancestor in the genus *Cyclotella*

**DOI:** 10.1186/1471-2148-11-125

**Published:** 2011-05-14

**Authors:** Andrew J Alverson, Bánk Beszteri, Matthew L Julius, Edward C Theriot

**Affiliations:** 1 Indiana University, Department of Biology, 1001 East Third Street, Bloomington, IN 47405, USA; 2 Alfred Wegener Institute for Polar and Marine Research, Am Handelshafen 12, 27570 Bremerhaven, Germany; 3 St. Cloud State University, Department of Biological Sciences, 720 Fourth Avenue South, St. Cloud, MN 56301, USA; 4 The University of Texas at Austin, Texas Natural Science Center, 2400 Trinity Street, Austin, TX 78705, USA

**Keywords:** *Cyclotella nana*, diatom, freshwater, marine, model species, *Thalassiosira pseudonana*

## Abstract

**Background:**

Publication of the first diatom genome, that of *Thalassiosira pseudonana*, established it as a model species for experimental and genomic studies of diatoms. Virtually every ensuing study has treated *T. pseudonana *as a marine diatom, with genomic and experimental data valued for their insights into the ecology and evolution of diatoms in the world's oceans.

**Results:**

The natural distribution of *T. pseudonana *spans both marine and fresh waters, and phylogenetic analyses of morphological and molecular datasets show that, 1) *T. pseudonana *marks an early divergence in a major freshwater radiation by diatoms, and 2) as a species, *T. pseudonana *is likely ancestrally freshwater. Marine strains therefore represent recent recolonizations of higher salinity habitats. In addition, the combination of a relatively nondescript form and a convoluted taxonomic history has introduced some confusion about the identity of *T. pseudonana *and, by extension, its phylogeny and ecology. We resolve these issues and use phylogenetic criteria to show that *T. pseudonana *is more appropriately classified by its original name, *Cyclotella nana*. *Cyclotella *contains a mix of marine and freshwater species and so more accurately conveys the complexities of the phylogenetic and natural histories of *T. pseudonana.*

**Conclusions:**

The multitude of physical barriers that likely must be overcome for diatoms to successfully colonize freshwaters suggests that the physiological traits of *T. pseudonana*, and the genes underlying those traits, might differ from those of strictly marine diatoms. The freshwater ancestry of *T. pseudonana *might therefore confound generalizations about the physiological and metabolic properties of marine diatoms. The freshwater component of *T. pseudonana*'s history merits careful consideration in the interpretation of experimental data collected for this important model species.

## Background

Diatoms are unicellular photosynthetic algae with secondary, red-algal-derived plastids [1]. With total diversity estimates in the tens to hundreds of thousands of species, diatoms are one of the most diverse lineages of eukaryotes [2] and are critically important to the ecology of both marine and fresh waters. Marine diatoms alone account for roughly one-fifth of global net primary production [3]. Efforts to understand the ecology and evolution of diatoms were catapulted forward when the first diatom genome, from a marine strain of *Thalassiosira pseudonana *(Hustedt) Hasle et Heimdal, was published in 2004 [4]. The genome revealed unanticipated metabolic pathways and gave a first glimpse into the mosaic nature of diatom nuclear genomes, which contain a mix of genetic material from the stramenopile host cell, diverse bacterial donors, and a succession of green and red algal endosymbionts [4-6].

In addition to its small (32 Mb) genome size, *T. pseudonana *was chosen for genome sequencing because it is generally considered representative of the large, widespread and predominantly marine genus *Thalassiosira *[4,7]. Although *T. pseudonana *has long been used in experimental studies [8-10], its genome sequence established it as the premier model for genome-enabled diatom research [11,12] on topics ranging from nutrient storage and metabolism [13-15] to silica biomineralization [16-19]. Data from these studies are often valued for their insights into the ecology and evolution of marine diatoms and, in turn, their determinant roles in the biogeochemical cycling of nutrients in the world's oceans [12]. Many of these inferences rest on the important assumption that *T. pseudonana *is a representative model for marine diatoms [20]. We summarize the phylogenetic and natural histories of *T. pseudonana *and show that it most likely descended from a freshwater ancestor. This portion of its history may have shaped several important physiological traits and the genes underlying those traits, confounding extrapolations to the biology of strictly marine diatoms. Finally, its relatively nondescript form and convoluted taxonomic history have introduced some confusion about the identity of *T. pseudonana *and, by extension, its phylogenetic and ecological histories. We resolve these issues and use phylogenetic criteria to show that *T. pseudonana *is more appropriately classified by its original name, *Cyclotella nana *Hustedt.

## Results

### The phylogeny and evolutionary ecology of *Thalassiosira pseudonana*

**Figure 1:**
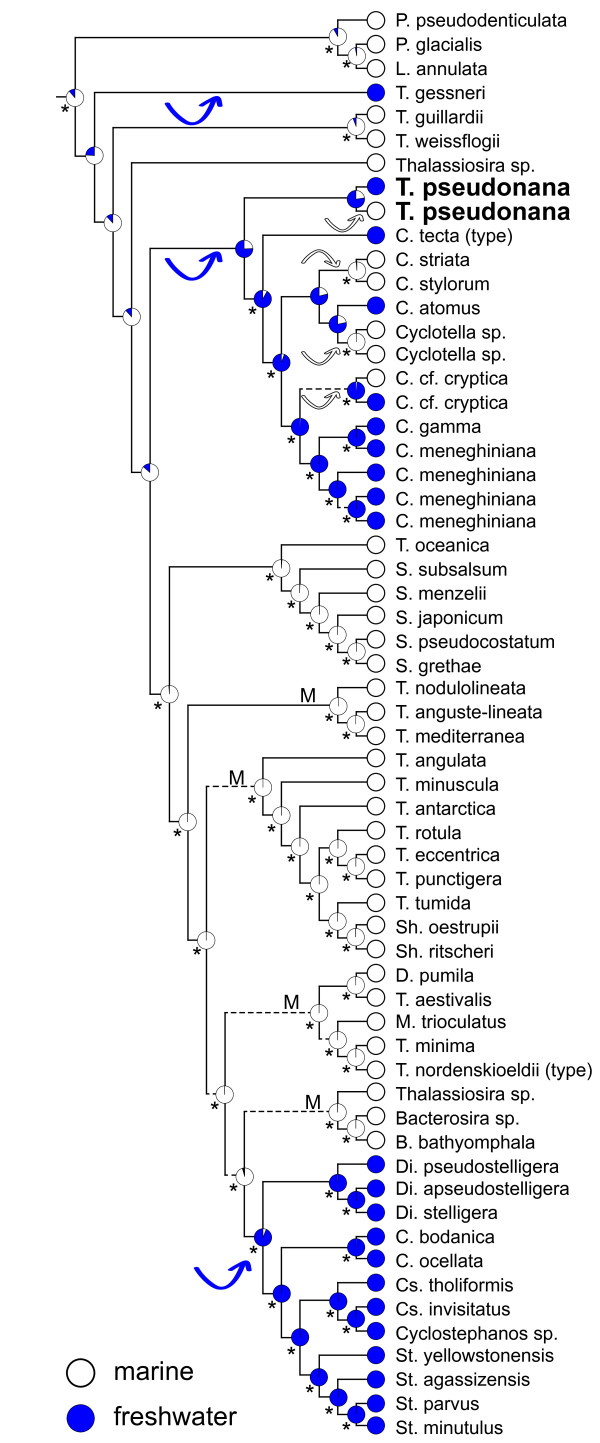
**Phylogeny of the diatom order Thalassiosirales based on a Bayesian analysis of four concatenated genes**. Pie graphs at internal nodes show the relative maximum likelihood support for the inferred ancestral habitat type, and asterisks show that the more strongly supported ancestral habitat type is significantly better than the alternative. Arrows represent inferred marine-to-freshwater (blue) and freshwater-to-marine (white) colonization events. Dashed lines identify nodes with <0.95 Bayesian posterior probability support, and lineages containing potentially strong candidates for alternative model marine diatoms are marked with "M." *Cyclotella tecta *and *Thalassiosira nordenskioeldii *are the nomenclatural types ("type") for their respective genera. Genus abbreviations are: *Bacterosira *(B), *Cyclotella *(C), *Cyclostephanos *(Cs), *Detonula *(D), *Discostella *(Di), *Lauderia *(L), *Minidiscus *(M), *Porosira *(P), *Shionodiscus *(Sh), *Skeletonema *(S), *Stephanodiscus *(St), and *Thalassiosira *(T). The figure is modified from ref. [25].

A combined phylogenetic analysis of two chloroplast (*psbC *and *rbcL*) and two nuclear (*SSU *and partial *LSU *rDNA) genes strongly and unambiguously resolved the phylogenetic position of *T. pseudonana *as sister to a clade of marine and freshwater species in the genus *Cyclotella *(Figure 1). This clade includes *C. meneghiniana*, which has been cited as an exemplar cosmopolitan freshwater diatom [21], and *C. tecta*, which is the nomenclatural type of the genus *Cyclotella *(Figure 1). The phylogeny also shows that members of the genus *Thalassiosira *are distributed across 10 different lineages within the Thalassiosirales (Figure 1). Among these, the lineages that include *T. pseudonana *and *T. nordenskioeldii *(the nomenclatural type of the genus *Thalassiosira*) are only distantly related (Figure 1).

**Figure 2:**
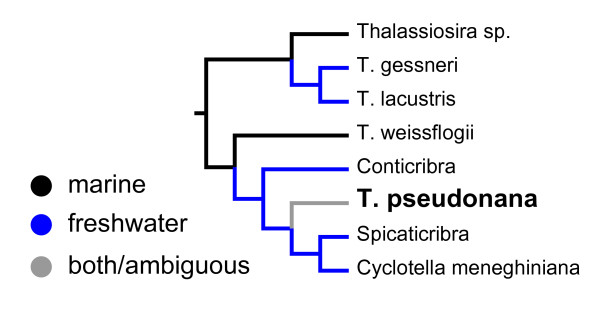
**Phylogenetic analysis of morphological characters places the model marine diatom *Thalassiosira pseudonana *within a clade of freshwater diatoms**. Ancestral states were reconstructed using parsimony. The full tree is shown in Additional File 1.

We extended a previous morphologically based phylogenetic analysis of Thalassiosirales [22] to include *T. pseudonana, C. meneghiniana*, and two recently described genera, *Conticribra *and *Spicaticribra *[23,24], both of which have morphological features that suggest a close relationship to the *T. pseudonana*+*C. meneghiniana *clade. The full morphological tree is shown in Additional File 1. Like the molecular phylogeny, morphological data also resolve *T. pseudonana *into a clade with *C. meneghiniana *(Figure 2). *Conticribra tricircularis*, which is known only from freshwaters [24], is sister to this clade (Figure 2).

Molecular phylogenetic analyses included both marine and freshwater strains of *T. pseudonana*, and these always formed a strongly supported clade [25]. The close relationship of these strains to *T. pseudonana *strain CCMP1335, which had its genome sequenced [4], is supported by their near-identical chloroplast *psbC *and *rbcL *genes and nuclear *SSU *and partial *LSU *rDNA genes (Additional file 2). Maximum likelihood reconstructions show that the common ancestor of the clade that includes *T. pseudonana*,* C. meneghiniana*, and *C. tecta *was most likely a freshwater diatom (Figure 1). Although the proportional likelihood strongly supports this conclusion (0.76 vs. 0.24), the difference is not statistically significant (Figure 1). However, morphological data place the monotypic freshwater genus *Conticribra *as sister to the *T. pseudonana*+*C. meneghiniana *clade, which unambiguously optimizes the common ancestor of the *T. pseudonana*+*C. meneghiniana *clade as freshwater in this analysis (Figure 2). Finally, the common ancestor of the two *T. pseudonana *strains was likely freshwater as well (Figure 1).

### The identity of *Thalassiosira pseudonana*

The combination of a relatively nondescript morphology and a series of three name changes over the years [26-29] has led to some uncertainty about the identity of *T. pseudonana *(Additional file 3). Several species are frequently confused with *T. pseudonana*, and two of these, *T. guillardii *and *T. oceanica*, were initially included under a broader concept of *T. pseudonana *(Additional file 4; ref. [30]). Resolution of these taxonomic and nomenclatural uncertainties required observation of the original sample (the "type material") from which *T. pseudonana*—originally named *Cyclotella nana*—was described (Additional file 5; ref. [29]).

**Figure 3:**
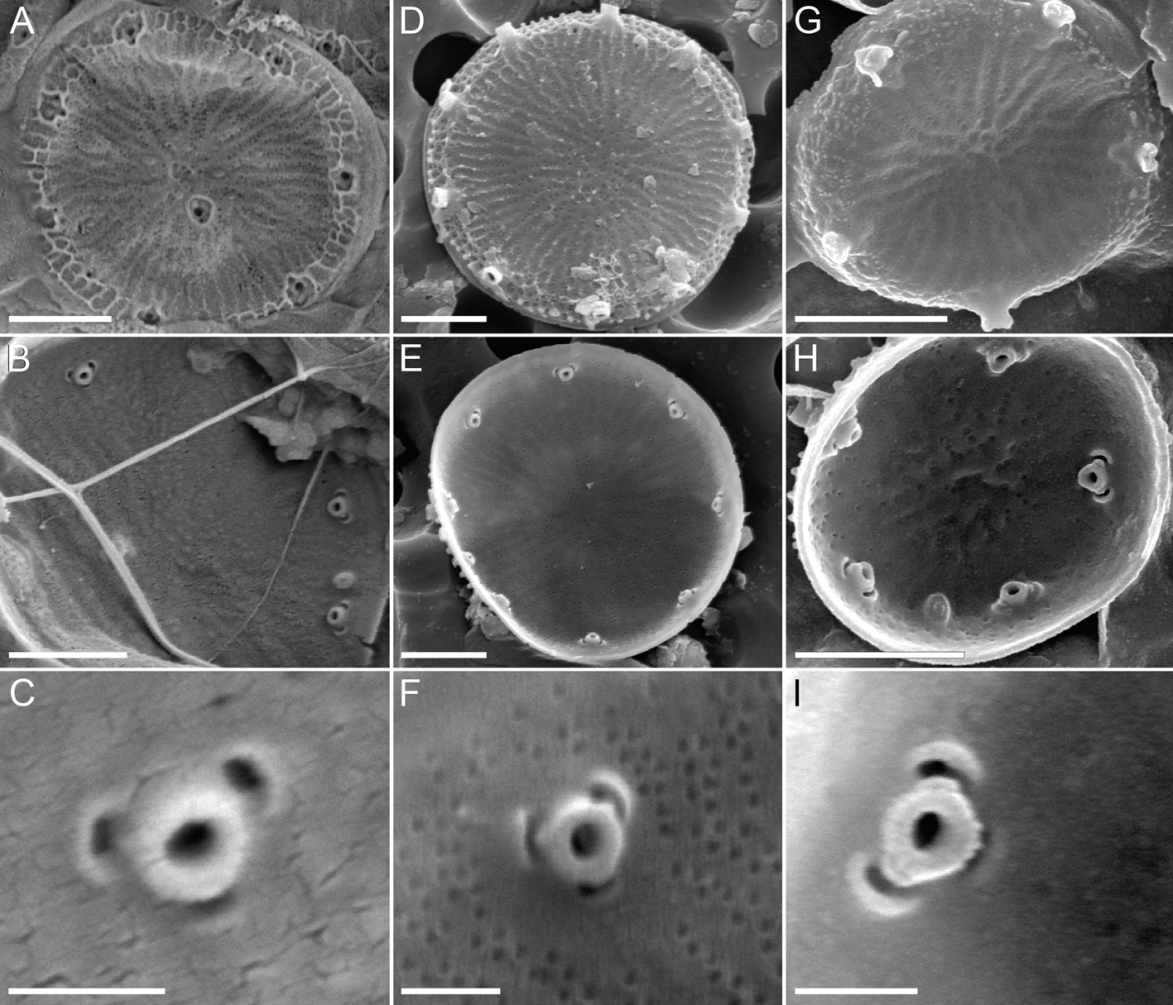
**Scanning electron micrographs of three ecologically diverse representatives of *Thalassiosira pseudonana***. (**A-C**) Marine culture strain CCMP1335 whose genome was sequenced; (**D-F**) freshwater specimens from the original type collection for the species from the River Wümme, Germany; (**G-I**) freshwater culture strain ETC1 from Lake Erie, Michigan, used in molecular phylogenetic analyses (Figure 1). The first and second rows show the cell exterior and interior, respectively (scale bar = 1 μm), and the third row shows the interior ultrastructure of the strutted process (scale bar = 200 nm).

Several small Thalassiosirales were found in the *C. nana *type material (Additional file 6), but the most common small diatom encountered matches both the original description of *C. nana *[29] and the traditional concept of *T. pseudonana *(Figure 3; refs. [28-34]). All of these diatoms, and those used for phylogenetic reconstruction (Figure 1) and genome sequencing [4], are small (less than 10 μm diameter), circular, radially symmetric diatoms with 0-3 central-area strutted processes, numerous marginal-area strutted processes with three satellite pores each, and a labiate process situated between two marginal-area strutted processes (Figure 3 and Additional file 7). Ultrastructural features of the strutted processes are phylogenetically informative [25], and the strutted processes of all these diatoms have prominent, raised cowlings around the satellite pores and a more-or-less prominent siliceous ridge (opercle) on the central strutted process tube, situated directly above the corresponding satellite pore (Figure 3 and Additional file 7).

Taken together, molecular and morphological evidence lead us to conclude that the model *T. pseudonana *strain CCMP1335 [4] and the culture strains used in molecular phylogenetic analyses (Figure 1) are all conspecific with specimens originally described as *Cyclotella nana *(Additional file 4).

## Discussion

### Robustness of phylogenetic results and ancestral reconstructions

Our conclusions about the ancestry of *T. pseudonana *rest primarily on the assumption that our analyses have accurately resolved its position within the broader Thalassiosirales phylogeny. As suggested by the numerous taxonomic and nomenclatural uncertainties (Additional files 3 and 4), *T. pseudonana *has gross morphological similarities to species of *Thalassiosira*, *Cyclotella*, and *Discostella*. In fact, *T. pseudonana *is commonly confused with several species of *Discostella *and *Thalassiosira *(viz., the purely freshwater species *D. pseudostelligera *and the purely marine species *T. guillardii *and *T. oceanica*). We included each of these species in our molecular dataset, and they all resolved well away from *T. pseudonana *(Figure 1). *Cyclotella *and *Thalassiosira *are especially large and morphologically diverse genera, though years of monographic research have led to numerous informally recognized morphological groups within each of them [34-43]. Our molecular dataset includes at least one representative from each of the informally designated groups within both *Thalassiosira *and *Cyclotella*. By sampling much of the known range of morphological diversity within these two genera, we have included many of the lineages within the Thalassiosirales phylogeny where one might predict *T. pseudonana *to fall.

The molecular dataset ultimately resolved *T. pseudonana *as sister to a clade that includes the *C. meneghiniana *species complex and the nomenclatural type of the genus *Cyclotella*, *C. tecta*. Comparing phylogenies derived from morphological and molecular datasets is a powerful way to detect phylogenetic incongruence, or likewise, to strengthen an existing phylogenetic hypothesis based on just one of the two data types [44]. Despite their different and largely non-overlapping taxon sampling schemes, the molecular and morphological datasets were congruent in their placement of *T. pseudonana *as sister to *Cyclotella *(Figures 1 and 2). Moreover, there was also congruence between morphology and molecules for the larger set of taxa common to both datasets, viz: (*T. gessneri*, (*T. weissflogii*, (*T. pseudonana*, *C. meneghiniana*))).

Our habitat codings were based on the habitat from which each culture originally was isolated. Although an individual culture strain might not capture the full range of genetic and phenotypic variability of its species, coding a culture strain by its natural habitat is conceptually similar to the common practice of representing a species in phylogenetic analyses with gene sequences from a single individual. *Thalassiosira pseudonana *can tolerate a wide range of salinities [30], so we could have coded each of the strains in our analyses as simply "euryhaline." For our purposes, however, individually coding each strain according to its natural habitat should have been more conservative, in that it allowed for a marine ancestral state reconstruction for both *T. pseudonana *and the nodes immediately surrounding it.

Although future data might show that some of the *T. pseudonana *strains considered here represent different biological species, it is not clear that those species would necessarily resolve along marine-freshwater lines. The finding by Guillard and Ryther [30] that three different *T. pseudonana *clones (3H, 5A and e.p.) maintained high growth rates from 0.5-37‰ salinity supports the hypothesis that *T. pseudonana *is, in fact, a single species that tolerates a wide range of salinities. Finally, the reconstruction of a freshwater ancestor for *T. pseudonana *(Figure 1) applies regardless of whether marine and freshwater strains are considered the same, or different, species.

To summarize, phylogenetic analyses place *T. pseudonana *as sister to a predominately freshwater clade of diatoms in the genus *Cyclotella*. The congruence between the molecular and morphological datasets provides especially strong support for this result [44]. Ancestral state reconstructions showed that the common ancestor of this clade was either unequivocally freshwater (morphological data, parsimony analysis) or likely freshwater (molecular data, likelihood analysis). Like any hypothesis, the results are potentially subject to change as new data become available or different character codings are used, but with respect to *T. pseudonana*, phylogenetic results and ancestral state reconstructions appear to be at least moderately robust to taxon sampling, data type, and optimality criterion.

### Is *Thalassiosira pseudonana *a good model for marine diatoms?

The tremendous importance of marine diatoms in global carbon fixation and marine food webs understandably compelled Armbrust et al. [4] to choose a marine diatom as the first for whole-genome sequencing [7]. *Thalassiosira pseudonana *belongs to the large and predominantly marine genus *Thalassiosira *and so was reasonably considered representative of that genus and of marine diatoms more broadly [7]. Subsequent phylogenetic analyses showed, however, that members of the genus *Thalassiosira *are spread across some 10 distinct evolutionary lineages (Figure 1). The extent of independent, unshared evolutionary history distinguishing these 10 lineages from one another is such that no single model species can possibly capture all of the diverse ecological, metabolic, and genomic properties of "*Thalassiosira*"—a prediction increasingly borne out by experimental and genomic data [45-47]. Of course, no one would expect uniformity across such a broad range of species diversity, but their shared classification in the genus *Thalassiosira *falsely suggests that the taxa in these 10 lineages constitute a more biologically coherent group than the phylogeny reveals them to be (Figure 1). The persistence of this, or any, misinformative classification represents a failure by systematists—ourselves included—to provide a phylogenetically based, and therefore biologically informative, alternative. Such an alternative would facilitate, for example, ecologically based selection of model species.

Salinity preference provides a glaring example of a trait that varies considerably across the 10 lineages of *Thalassiosira*, and Thalassiosirales as a whole (Figure 1). With a distribution that spans both marine and freshwater habitats, *T. pseudonana *embodies this variation. The evolutionary history of Thalassiosirales includes just a few major freshwater diversifications [25], and *T. pseudonana *marks an early divergence in one of them (Figure 1). In fact, phylogenetic analyses and formal mapping of habitat preference onto phylogenetic trees show that, 1) *T. pseudonana *is part of a large clade that is likely ancestrally freshwater (Figures 1 and 2), and 2) as a species, *T. pseudonana *is likely ancestrally freshwater as well (Figure 1). If so, these data suggest that marine strains of *T. pseudonana *represent recent recolonizations of higher salinity habitats by a derived freshwater ancestor (Figures 1 and 2).

Parker et al. [20] keenly raised the question as to how well our current selection of model microalgae represent their corresponding dominant forms in the marine environment. The data presented here suggest that the substantial freshwater component of *T. pseudonana*'s phylogenetic history might confound its use of as a model for marine diatoms. For example, there is great interest in understanding how diatoms sequester, store, and use iron because it limits their growth over vast offshore regions of the world's oceans [14,15,46,47]. By contrast, iron generally does not limit diatom growth in freshwater [48] and coastal ecosystems [49]. Experimental data show that coastal marine strains of *T. pseudonana *and *T. weissflogii *require more iron to maintain a maximal growth rate than a marine strain of the pennate diatom, *Phaeodactylum tricornutum*, and the oceanic diatom, *T. oceanica*, respectively [14,47]. As currently understood, these differences reflect either vastly different iron uptake [14] and storage [15] architectures between entirely different classes of diatoms (pennates vs. non-pennates), or slight modifications of shared iron uptake [46] or photosynthetic [47] architectures between closely related species. Some of these differences might, at least in part, reflect that *T. pseudonana *descended from a freshwater ancestor that experienced different (perhaps relaxed) constraints on its iron uptake and utilization machinery. Extending studies like that of Kustka et al. [14] to include strictly freshwater pennate and non-pennate diatoms along with strictly marine pennate and non-pennates might help tease apart the relative importance of shared history versus shared ecology in the evolution of iron metabolism in diatoms. In this context, "strictly" refers to diatoms that occur exclusively in either marine or freshwaters and whose immediate ancestor shared a similar distribution (e.g., Figure 1, "M").

Among the several hundred species of Thalassiosirales, which might provide a more representative model for marine diatoms? The phylogeny provides a powerful, predictive guide for selecting candidates. In this case, one might first consider the five strictly marine clades (Figure 1, "M") and then winnow down the candidate pool based on practical (e.g., genome size and amenability to cell culture) and ecological (e.g., oceanic vs. offshore) criteria.

### What is *Thalassiosira pseudonana*?

The diatom originally described as *Cyclotella nana *from the River Wümme has also gone by the names *T. pseudonana *[28], *C. pseudonana *[27], and most recently, *Discostella nana *[26] (Additional file 3). Like grammar, common usage tends to prevail, and this diatom became universally known as *T. pseudonana*. Importantly, none of the name changes was based on direct observations of the type material, and none either had [27,28] or took advantage of [26] the insights of a strongly supported phylogenetic hypothesis for the Thalassiosirales [25]. A combination of evidence (Figure 3, Additional files 3 and 4) shows that the diatom originally described by Hustedt [29] as *C. nana *corresponds to the traditional concept of *T. pseudonana *[29-34]. Furthermore, phylogenetic analyses show that *T. pseudonana *is part of a strongly supported clade that includes *C. tecta*, which is the nomenclatural type of the genus *Cyclotella *(Figure 1). By contrast—short of creating a genus that includes nearly the whole of Thalassiosirales—a genus that would include both *T. pseudonana *and *T. nordenskioeldii *(the type species of *Thalassiosira*) cannot be reconciled with the phylogeny (Figure 1). We are therefore compelled to resurrect the original name of *T. pseudonana*, *Cyclotella nana *[29], and deprecate *T. pseudonana *[28], *C. pseudonana *[27], and *D. nana *[26] as synonyms.

## Conclusions

Publication of the *T. pseudonana *genome established this species as a model for experimental and genomic studies of diatoms [4], virtually all of which treat *T. pseudonana *as a marine diatom. Given the central roles of marine diatoms in global primary production, marine food webs, and the biogeochemical cycling of important nutrients, there is much to gain in understanding how marine diatoms interact with their environment. This understanding will come in large part from the development and study of model organisms [20] whose traits invariably have been shaped by their phylogeny. Phylogenetic analyses of both morphological and molecular data show that *T. pseudonana *likely descended from a freshwater ancestor. In some cases, this unusual history might confound extrapolations to strictly marine diatoms. Interestingly, the other diatom with a fully sequenced genome, *P. tricornutum*, also occurs in freshwater, brackish and marine habitats [50] and so might not be an ideal ecological model for marine diatoms either [20]. Additional genomes from ecologically diverse diatoms will bear these issues out and show, for example, whether the peculiar evolutionary histories of *T. pseudonana *and *P. tricornutum *account for some portion of the unusually large genomic divergence between the two species [6].

With few exceptions, diatoms are generally classified into genera that are uniformly marine or freshwater in distribution [51]. Although originally described from a predominantly freshwater river and classified in what is traditionally considered a freshwater genus (*Cyclotella*), an unfortunate series of nomenclatural changes ultimately landed *T. pseudonana *in a predominantly marine genus. Although the full extent to which *T. pseudonana*'s name overshadowed the nuances of its ecology is a matter of speculation, *T. pseudonana *highlights the continued importance of taxonomy and systematics in the post-genomic era and echoes a view espoused by Linnaeus some 260 years ago in his landmark botanical text, Philosophia Botanica: "If you do not know the names of things, the knowledge of them is lost too." Beyond its name, a better appreciation of the complex phylogenetic and natural histories of *T. pseudonana *has the potential to shape and improve our understanding of diatom ecology and evolution more generally.

## Methods

We examined isolectotype material for *C. nana *(= *T. pseudonana*) to resolve several taxonomic and nomenclatural uncertainties (Additional files 3, 4 and 5). The sample is sediment collected by Friedrich Hustedt [29] from the River Wümme near Bremen, Germany, and corresponds to lectotype slide BRM 380/36 from the diatom collection at the Alfred Wegener Institute (Additional file 5).

All diatom samples were soaked in 30% hydrogen peroxide overnight to dissolve organic matter, then rinsed with distilled water several times and concentrated onto filter pads. Dried filter pads were affixed to scanning electron microscope (SEM) stubs with double-sided adhesive carbon discs, and coated with 15 nm of Iridium using a Cressington 208 Bench Top Sputter Coater. SEM observations were made with a Zeiss Supra 40 VP.

Alverson et al. [25] used two chloroplast (*psbC *and *rbcL*) and two nuclear genes (*SSU *and partial *LSU *rDNA) to reconstruct the phylogeny of Thalassiosirales. The 82-taxon matrix was nearly complete, with one of the four genes missing for just three taxa [25]. The final tree (Figure 1) was based on a Bayesian analysis of all four concatenated genes (5,102 characters total). The protein-coding *psbC *and *rbcL *genes were treated as a single marker, with first and second codon positions (GTR + G + I) modeled separately from third codon positions (GTR + G + I) [52]. Separate GTR + G + I models were applied to the *LSU *rDNA gene and *SSU *rDNA loops, and the Doublet + G + I model was applied to the *SSU *rDNA stems [25]. Additional information about the data matrix, partitions, and exact model specifications can be found in the publicly available data file (identifier doi:10.5061/dryad.8661 at http://datadryad.org/). We performed four independent Bayesian runs, each of which used four Markov chains and was run for a total 40 million generations. The *slide *and *cump *analyses in the AWTY diagnostic software tool [53] were used to assess the stationarity of each run, and the AWTY *compare *analysis was used to determine whether the stationary phases of the four independent runs had sampled from the same posterior distribution. The latter analysis showed that that one of the four runs sampled a slightly different posterior distribution than the other three runs, so the final consensus tree (Figure 1) was based on the combined posterior distributions of the three similar runs.

Habitat (marine or freshwater) was scored based the natural provenance of the culture. The collection site for each culture is listed in Alverson et al. [25]. We scored strains collected from brackish and other low-salinity habitats as marine, thus biasing towards marine rather than freshwater ancestral reconstructions (all other things been equal). Maximum likelihood reconstructions used the Asymmetrical Markov k-state 2-parameter model as implemented in the Mesquite software package.

Several new freshwater species with gross morphological similarity to *T. pseudonana *and relatives have been described [23,24] since the publication of Alverson et al. [25]. We added these taxa to the morphological data matrix of Julius and Tanimura [22], and used the branch and bound search algorithm in PAUP* to find the most parsimonious trees for this matrix. Character descriptions are available in Julius and Tanimura [22]. All data matrices are available from Dryad (http://datadryad.org/) using identifiers doi:10.5061/dryad.8661 and doi:10.5061/dryad.8663.

## Authors' contributions

AJA conceived the study, performed phylogenetic analyses, captured and analyzed SEM images, and drafted the manuscript. BB gathered, translated, and interpreted historical data on *Cyclotella nana *and helped in the interpretation of all results. MLJ gathered the morphological data used for phylogenetic analyses and helped in the interpretation of all results. ECT participated in study design and coordination, helped capture and analyze SEM images, and helped in the interpretation of all results. All authors read and approved the final manuscript.

## Supplementary Material

Additional file 1 Strict consensus of eight most parsimonious trees from a phylogenetic analysis of 32 morphological characters for select Thalassiosirales. Click here for file

Additional file 2 Pairwise differences among *Thalassiosira pseudonana *culture strains at four loci. Click here for file

Additional file 3 The nomenclatural history of *Thalassiosira pseudonana*. Click here for file

Additional file 4 The identity of *Thalassiosira pseudonana*. Click here for file

Additional file 5 Hustedt's observations on the type locality of *Cyclotella nana *(= *T. pseudonana*). Click here for file

Additional file 6 Scanning electron micrographs of thalassiosiroid diatoms in the type material (Additional file 4) of *Cyclotella nana *(= *Thalassiosira pseudonana*): *Stephanodiscus hantzschii *Grunow (**A**, **B**), *Stephanodiscus minutulus *(Kützing) Cleve & Möller (**C**, **D**), *Discostella pseudostelligera *(Hustedt) Houk & Klee (**E**-**H**), *Cyclotella striata *(Kützing) Grunow in Cleve & Grunow (**I**), *Cyclotella atomus *Hustedt (**J**), *Cyclotella meneghiniana *Kützing (**K**,**L**), *Cyclostephanos invisitatus *(Hohn & Hellerman) Theriot, Stoermer & Håkansson (**M**), *Shionodiscus *sp. (**N**), *Thalassiosira *sp. 1 (**O**), *Thalassiosira *sp. 2 (**P**). Scale bar = 2 μM. Click here for file

Additional file 7 **Scanning electron micrographs showing exterior (**A**, **B**) and interior (**C**, **D**) views of *Thalassiosira pseudonana *(marine strain NEPC709) from Alverson et al. **[25]. Scale bar = 2 μM. Click here for file
