# Supplementary figures and images for: The model marine diatom Thalassiosira pseudonana likely descended from a freshwater ancestor in the genus Cyclotella
Source: BMC Evol Biol. 2011 May 14;11:125. doi: 10.1186/1471-2148-11-125 (PMC3121624; doi:10.1186/1471-2148-11-125)

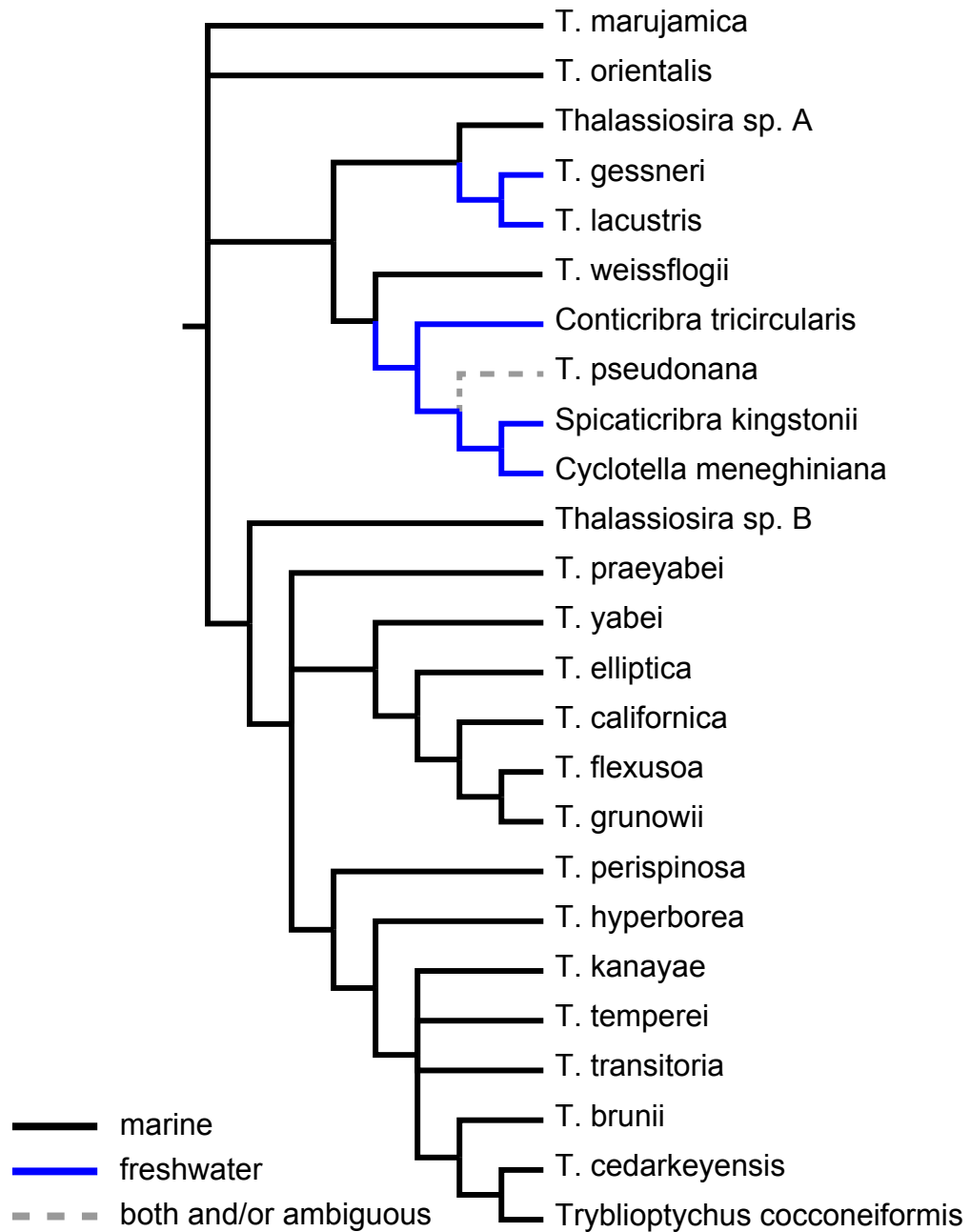

Supplement: Additional file 1 — Strict consensus of eight most parsimonious trees from a phylogenetic analysis of 32 morphological characters for select Thalassiosirales. [file 1471-2148-11-125-S1.PDF]

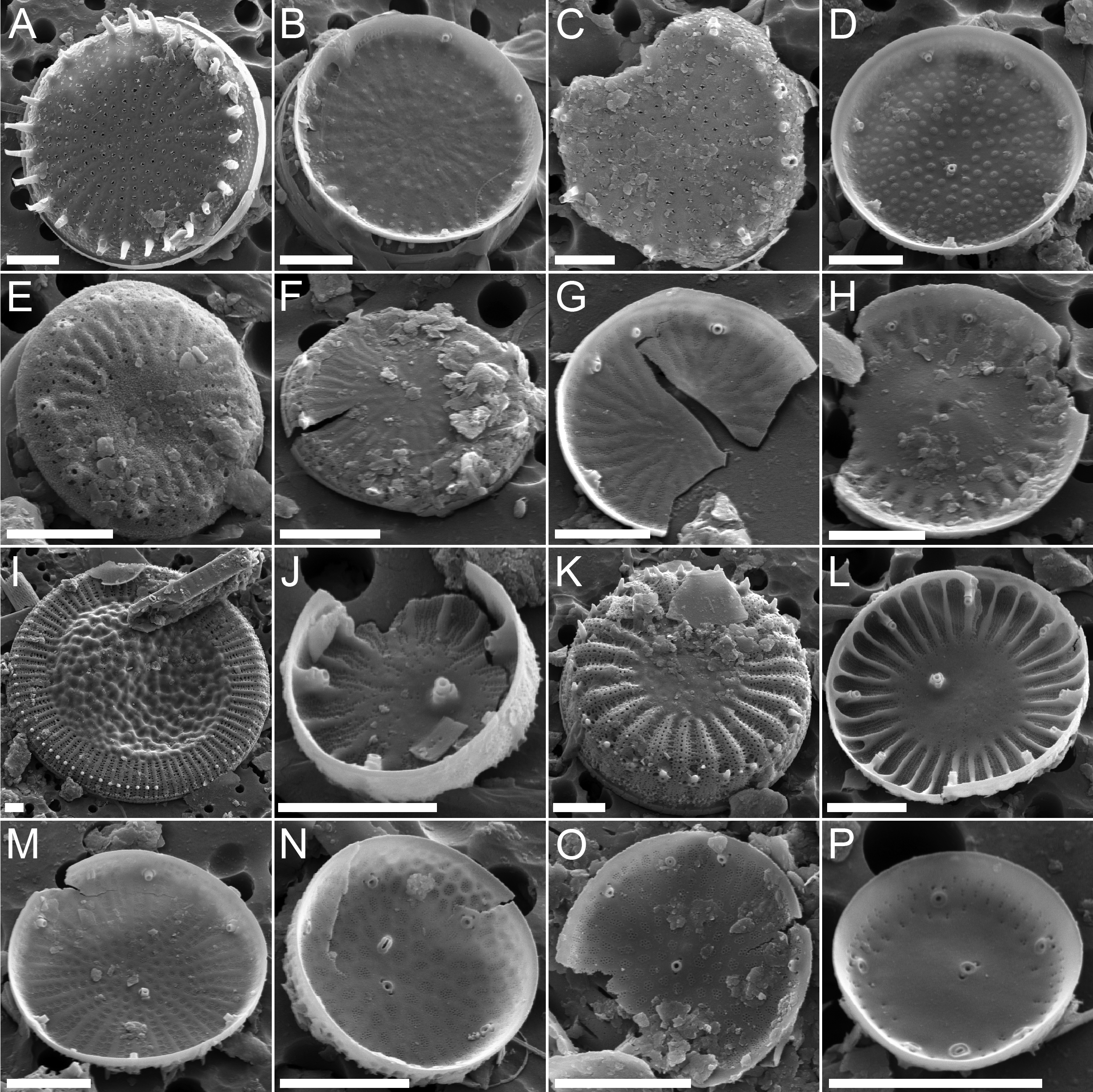

Supplement: Additional file 6 — Scanning electron micrographs of thalassiosiroid diatoms in the type material (Additional file 4) of Cyclotella nana (= Thalassiosira pseudonana): Stephanodiscus hantzschii Grunow (A, B), Stephanodiscus minutulus (Kützing) Cleve & Möller (C, D), Discostella pseudostelligera (Hustedt) Houk & Klee (E-H), Cyclotella striata (Kützing) Grunow in Cleve & Grunow (I), Cyclotella atomus Hustedt (J), Cyclotella meneghiniana Kützing (K,L), Cyclostephanos invisitatus (Hohn & Hellerman) Theriot, Stoermer & Håkansson (M), Shionodiscus sp. (N), Thalassiosira sp. 1 (O), Thalassiosira sp. 2 (P). Scale bar = 2 μM. [file 1471-2148-11-125-S6.TIFF]

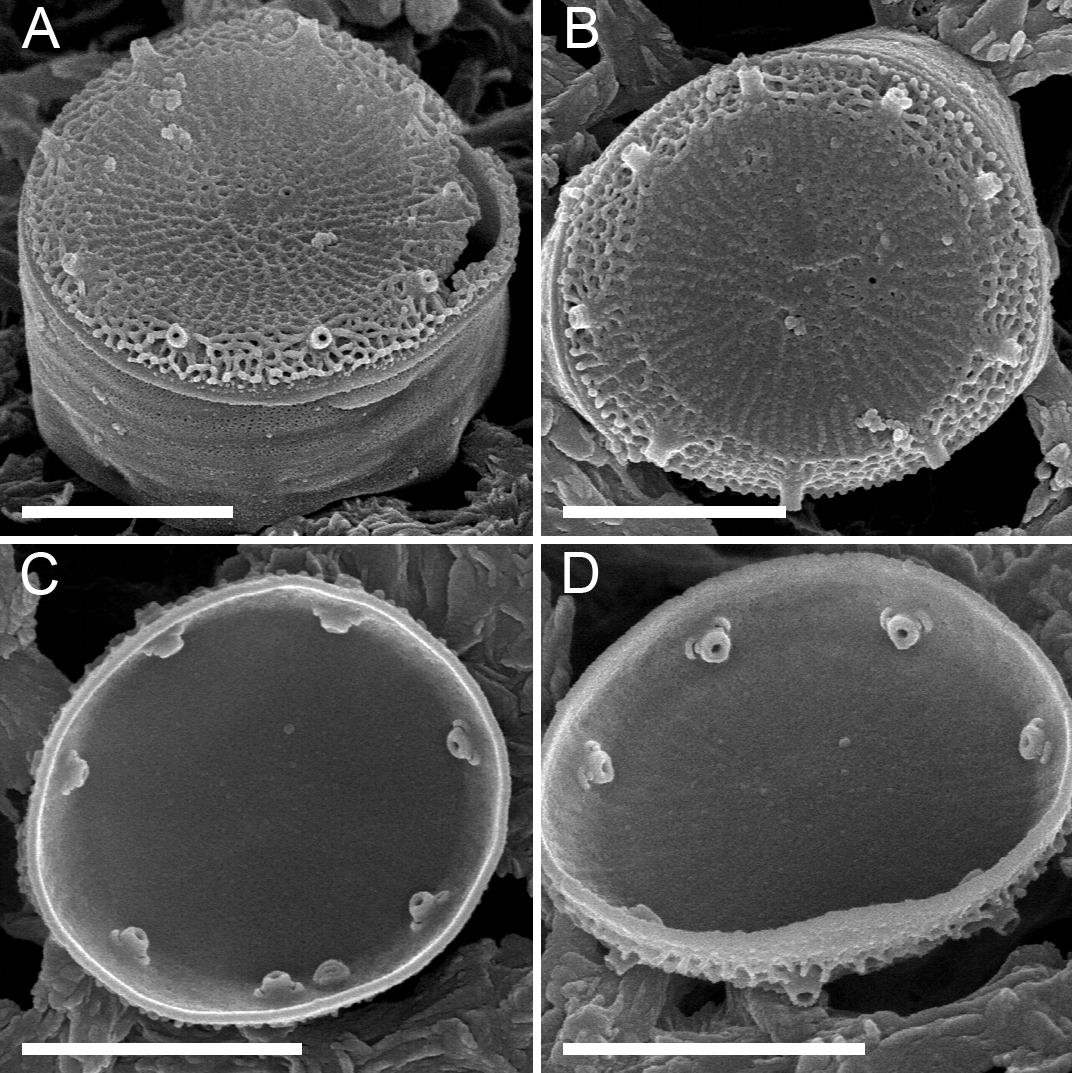

Supplement: Additional file 7 — Scanning electron micrographs showing exterior (A, B) and interior (C, D) views of Thalassiosira pseudonana (marine strain NEPC709) from Alverson et al. [25]. Scale bar = 2 μM. [file 1471-2148-11-125-S7.TIFF]
