# Supplementary material for: The model marine diatom Thalassiosira pseudonana likely descended from a freshwater ancestor in the genus Cyclotella
Source: BMC Evol Biol. 2011 May 14;11:125. doi: 10.1186/1471-2148-11-125 (PMC3121624; doi:10.1186/1471-2148-11-125)
Supplement: Additional file 2 — Pairwise differences among Thalassiosira pseudonana culture strains at four loci. [file 1471-2148-11-125-S2.PDF]

Additional File 2. Pairwise differences among *Thalassiosira pseudonana* strains at four loci.

Table S1a. Number of pairwise differences in the nuclear *SSU* rDNA gene (~1750 nt) among different marine (M) and freshwater (F) culture strains of *T. pseudonana*.

***SSU* rDNA**	CCMP1335	CCMP1057	NEPC709	ETC1
(M) CCMP1335	—	**0**	**0**	**0**
(M) CCMP1057		—	**0**	**0**
(M) NEPC709			—	**0**
(F) ETC1				—

Table S1b. Number of pairwise differences in the nuclear *LSU* d1–d2 region (~550 nt) among different marine (M) and freshwater (F) culture strains of *T. pseudonana*.

***LSU* rDNA**	CCMP1335	CCMP1057	NEPC709	ETC1
(M) CCMP1335	—	**1**	**1**	**2**
(M) CCMP1057		—	**0**	**3**
(M) NEPC709			—	**3**
(F) ETC1				—

Table S1c. Number of pairwise differences in the chloroplast *rbcL* gene (~1473 nt) among different marine (M) and freshwater (F) culture strains of *T. pseudonana*.

rbcL	CCMP1335	CCMP1057	NEPC709	ETC1
(M) CCMP1335	—	**0**	**0**	**3**[a]
(M) CCMP1057		—	**0**	**3**[a]
(M) NEPC709			—	**3**[a]
(F) ETC1				—

[a]Two of these differences are involve base calls that are ambiguous in ETC1 (A/T) and unambiguous (A) in the other strains.

Table S1d. Number of pairwise differences in the chloroplast *psbC* gene (~1200 nt) among different marine (M) and freshwater (F) culture strains of *T. pseudonana*.

psbC	CCMP1335	CCMP1057	NEPC709	ETC1
(M) CCMP1335	—	**0**	**0**	**0**
(M) CCMP1057		—	**0**	**0**
(M) NEPC709			—	**0**
(F) ETC1				—
