# Supplementary material for: The model marine diatom Thalassiosira pseudonana likely descended from a freshwater ancestor in the genus Cyclotella
Source: BMC Evol Biol. 2011 May 14;11:125. doi: 10.1186/1471-2148-11-125 (PMC3121624; doi:10.1186/1471-2148-11-125)
Supplement: Additional file 3 — The nomenclatural history of Thalassiosira pseudonana. [file 1471-2148-11-125-S3.PDF]

Additional File 3. The nomenclatural history of *Thalassiosira pseudonana*.

Cyclotella nana Hustedt was described from plankton and sediment samples from the River Wümme and River Weser, but no type was selected [1]. Simonsen [2] selected Hustedt 380/36 as the lectotype, which traces to a sediment sample from the River Wümme. Hasle and Heimdal [3] transferred *C. nana* to *Thalassiosira* as *T. pseudonana* Hasle and Heimdal because the name *T. nana* (*T. nana* Lohmann) was occupied. Chang and Steinberg [4] created the superfluous nomenclatural synonym *Cyclotella pseudonana* (Hasle and Heimdal) Chang and Steinberg, based on *T. pseudonana* Hasle and Heimdal. Later, Chang and Chang-Schneider transferred *C. nana* to *Discotella* as *D. nana* Chang [5]. None of the name changes were based on direct observation of the *C. nana* type material.

1. Hustedt F: **Die Diatomeenflora des Flußsystems der Weser im Gebiet der Hansestadt Bremen [Diatom flora of the tributaries of the Weser near the city of Bremen]**. *Abh Naturw Ver Bremen* 1957, **34**:181-440.
2. Simonsen R: **Atlas and Catalogue of the Diatom Types of Friedrich Hustedt. Volume 3. Atlas, Plates 396-772**. Berlin: J. Cramer; 1987.
3. Hasle GR, Heimdal BR: **Some species of the centric diatom genus *Thalassiosira* studied in the light and electron microscopes**. *Beihefte zur Nova Hedwigia* 1970, **31**:543-581.
4. Chang TP, Steinberg C: **Identifizierung von nanoplanktischen Kieselalgen (Centrales, Bacillariophyceae) in der Rott und im Rott-Stausee (Bayern, Bundesrepublik Deutschland) [Identification of nanoplanktonic diatoms (Centrales, Bacillariophyceae) in River Rott and Rott Reservoir (Bavaria, F.R.G.)]**. *Arch Protistenkd* 1989, **137**(2):111-129.
5. Chang TP, Chang-Scheider H: **Zentrische kieselalgen in Kemptener seen [Centric diatoms in lakes of Kempten]**. *Ber Deut Bot Ges* 2008, **78**:5-15.
