# Supplementary material for: The model marine diatom Thalassiosira pseudonana likely descended from a freshwater ancestor in the genus Cyclotella
Source: BMC Evol Biol. 2011 May 14;11:125. doi: 10.1186/1471-2148-11-125 (PMC3121624; doi:10.1186/1471-2148-11-125)
Supplement: Additional file 4 — The identity of Thalassiosira pseudonana. [file 1471-2148-11-125-S4.PDF]

Additional File 4. The identity of *Thalassiosira pseudonana*

Previous concepts of *Thalassiosira pseudonana*

Observations from diverse literature sources reveal that diatomists have more-or-less consistently viewed *T. pseudonana* as a small (less than 10 µm diameter) centric diatom with 0–3 central area strutted processes, a relatively high number of marginal area strutted processes, and a labiate process situated roughly equidistantly between two marginal area strutted processes [1-7]. Ultrastructural features common to diatoms illustrated by these authors include three satellite pores per marginal area strutted process, the presence of more-or-less prominent cowlings around the strutted process satellite pores, and a more-or-less prominent siliceous ridge (opercle) on the central strutted process tube situated directly above each satellite pore. The labiate process is typically oval in shape.

Hasle [3] summarized these and other features of *T. pseudonana* and similar diatoms, particularly *T. guillardii* Hasle and *T. oceanica* Hasle, both of which were initially included under a broader concept of *T. pseudonana* [2]. *Thalassiosira guillardii* is distinguished from *T. pseudonana* by the position of the rimportula (which takes the place of a marginal strutted process in the marginal area process pattern, as opposed to being between two marginal strutted processes in *T. pseudonana*), the shape of the labiate process (rectangular versus oval), and by having four rather than three satellite pores per marginal area strutted process. *Thalassiosira oceanica* has roughly half as many marginal area strutted process as *T. pseudonana*, its labiate process is generally closer to a marginal strutted process, the labiate process shape is rectangular, and there are again four satellite pores per marginal strutted process.

Thalassiosira pseudonana appears virtually structureless in the light microscope (LM). In good preparations, in which all organic matter is removed and in which the valves are embedded in a high refractive index mounting medium, it is possible to see "apiculi" [5], the dots that represent the labiate and strutted processes. However, we have never been able to visualize fascicles or costae, nor have we have seen them illustrated. We believe that they would have been essentially invisible to Hustedt, and indeed that is how he both drew and described the species [5]:

"Zellen sehr zart, Schalen flach, kreisförmig, ohne erkennbare Struktur, am Rande mit wenigen, meistens 5 kleinen Dörnchen."

Our translation: "Cells very delicate, valves flat, circular, without observable structure, with a few, mostly 5, small spines."

Diatoms in the type material of *Cyclotella nana* (=*Thalassiosira pseudonana*)

Simonsen selected a specimen on slide 380/36 from the diatom collection at the Alfred-Wegener-Institut as the lectoype of *C. nana* and illustrated circled specimens on that slide [8]. The specimens illustrated by Simonsen [8] conform to the above description. We made slides of isolectotype material and found numerous small Thalassiosirales. Some of these resembled the description above, with marginal "apiculi" and no apparent structure on the valve face. Some specimens, however, had very faint costae with a clear central area, reminiscent of *Discostella* species, such as *D. stelligera* and *D. pseudostelligera* [9].

Many other small Thalassiosirales on the slide probably were distinguished easily by Hustedt and were probably known to him variously as *Stephanodiscus astraeae* var. *minutula* (now *S. minutulus*), *Stephanodiscus hantzschii* (perhaps including what is now known as *Cyclostephanos invisitatus*), and several species of *Cyclotella*, the smallest being *C. atomus* Hustedt. Although the latter attains the small size of *T. pseudonana* as generally conceived, it has prominent costae and was described by Hustedt himself in 1938. The species was therefore well known to him, making it unlikely that he would have confused it with *Cyclotella nana*.

Scanning electron microscope (SEM) observations revealed these species and more. We will focus on the diatoms with a simple valve structure that likely would have appeared hyaline or nearly so in the LM. We observed four morphotypes with such structure, which we consider in turn below. The most common of these corresponded well to the common concept of *T. pseudonana* in all respects.

***Shionodiscus* sp. (Additional file 6, N)—**We found a single specimen of a rare morphotype with areolae on the valve face and mantle (Additional file 6, N). Although areolae are distinct in this specimen, it is possible that they would not be visible in the LM due to their small size (areolar density is about 40 in 10 μm). The marginal area strutted processes are widely spaced, and each has three satellite pores that are partially occluded by a small tab, with no cowlings (Additional file 6, N). The single central area strutted process has two satellite pores (Additional file 6, N). The labiate process is on the valve face (Additional file 6, N), suggesting this is a species of *Shionodiscus* [10]. The labiate and central area strutted processes would likely be visible but indistinguishable in the LM. The common concept of *T. pseudonana* includes diatoms with as many as 2 central area strutted processes, so it is possible that *T. pseudonana* and this *Shionodiscus* species could be confused in the LM. However, the extreme rarity of *Shionodiscus* in this sample strongly suggests that it does not correspond to Hustedt's *C. nana*. We found no specimens that we could confidently identify as an external view of this species.

***Thalassiosira sp. 2* (Additional file 6, P)—**We found only one valve of the type illustrated in Additional file 6P, which we labeled "*Thalassiosira* sp. 2". Similar to *T. pseudonana*, the labiate process is situated roughly between two marginal area strutted processes in *Thalassiosira* sp. 2 (Additional file 6, P). However, several features distinguish the two species. In *Thalassiosira* sp. 2, the entire central area was hyaline, with distinct areolae restricted to the mantle (Additional file 6, P). The marginal area strutted processes were unusual in morphology, with a thick-walled central tube and what appeared to be only two small satellite pores situated below the central tube (Additional file 6, P). There were neither opercles occluding the pores nor cowlings surrounding the pores (Additional file 6, P). We found no specimens that we could confidently identify as the exterior of this morphotype.

***Discostella pseudostelligera* (Additional file 6, E–H)—** *Discostella pseudostelligera* is the only other diatom in this sample that could conceivably be confused with the common morphotype (Additional file 6, E–H). Like the common *T. pseudonana* form, *D. pseudostelligera* cells are small (3.4–5.4 μm diameter) in this sample (Additional file 6, E–H). In this sample, *D. pseudostelligera* has costae densities ranging from 20–32 per 10 μm circumference, suggesting the costae would be visible in LM. Our own LM observations of the isolectotype material showed that costae are clearly visible in most *D. pseudostelligera* specimens. Although some specimens of *D. pseudostelligera* are lightly silicified in this sample and the costae are sometimes faint, we find it unlikely that Hustedt would have mistaken this diatom for the common form, given that he himself described *D. pseudostelligera* some 20 years earlier [11]. In the SEM, marginal area strutted processes in *D. pseudostelligera* have two satellite pores rather than three as in the

common *T. pseudonana* form, and its internal morphology of the labiate process is distinctly circular, as opposed to the more oval morphology of the common form.

Cyclotella nana **(Fig. 3)—**We conclude that the morphology of the most common form represents *Cyclotella nana* Hustedt [=*Thalassiosira pseudonana* (Hustedt) Hasle et Heimdal]. Of the four morphotypes under consideration, the most common form outnumbered the other two by nearly two orders of magnitude, so it is most likely that Hustedt circled such a specimen on the lectotype slide. The SEM observations of isolectotype material also clearly show that the most common form conforms well to most observations of diatoms identified as *T. pseudonana* or *C. nana* in the literature [1-7].

Identity of *T. pseudonana* strain CCMP1335 and strains from Alverson et al. [12]

The three culture strains (ETC1, CCMP1057, and NEPC709) identified as *T. pseudonana* by Alverson et al. [12] formed a strongly supported clade in all analyses [12]. These three strains share identical or near-identical sequences at the two chloroplast and two nuclear loci used to reconstruct the phylogeny (Additional file 2). They also have identical or near-identical sequences to *T. pseudonana* strain CCMP1335, which was used for whole-genome sequencing [13] (Additional file 2).

Although CCMP1057 was not observed with SEM, the other two strains from Alverson et al. [12] and CCMP1335 fit the common form in the *C. nana* isolectotype material in all qualitative aspects (Fig. 3). Internally, the labiate process is oval, and the marginal area strutted processes have 3 satellite pores each, a prominent opercle on the central tube, and distinctly raised and thickened cowlings. ETC1 specimens lacked a central area strutted process, whereas NEPC709 and CCMP1335 specimens most often had a central area strutted process. These cultures were isolated from single cells and grown under different conditions and at different times, so it is impossible to determine if these morphological differences are genetically fixed with these data alone.

References

1. Belcher JH, Swale EMF: **Species of *Thalassiosira* (Diatoms, Bacillariophyceae) in the plankton of English rivers**. *Br Phycol J* 1977, **12**(3):291-297.
2. Guillard RRL, Ryther JH: **Studies of marine planktonic diatoms. I. *Cyclotella nana* Hustedt and *Detonula confervacea* (Cleve) Gran.** *Can J Microbiol* 1962, **8**:229-239.
3. Hasle GR: **The marine, planktonic diatoms *Thalassiosira oceanica* sp. nov. and *T. partheneia***. *J Phycol* 1983, **19**(2):220-229.
4. Hasle GR, Heimdal BR: **Some species of the centric diatom genus *Thalassiosira* studied in the light and electron microscopes**. *Beihefte zur Nova Hedwigia* 1970, **31**:543-581.
5. Hustedt F: **Die Diatomeenflora des Flußsystems der Weser im Gebiet der Hansestadt Bremen [Diatom flora of the tributaries of the Weser near the city of Bremen]**. *Abh Naturw Ver Bremen* 1957, **34**:181-440.
6. Hustedt F: **Die Diatomeenflora der Unterweser von der Lesummündung bis Bremerhaven mit Berücksichtigung des Unterlaufs der Hunte und Geeste [Diatom flora of the lower Weser from the Lesum estuary to Bremerhaven, with consideration of the lower Hunte and Geeste]**. *Veröff Inst Meeresforsch* 1959, **6**:13-176.

7. Lowe RL: **Comparative ultrastructure of the valves of some *Cyclotella* species (Bacillariophyceae)**. *J Phycol* 1975, **11**(4):415-424.
8. Simonsen R: **Atlas and Catalogue of the Diatom Types of Friedrich Hustedt. Volume 3. Atlas, Plates 396-772**. Berlin: J. Cramer; 1987.
9. Houk V, Klee R: **The stelligeroid taxa of the genus *Cyclotella* (Kützing) Brébisson (Bacillariophyceae) and their transfer into the new genus *Discostella* gen. nov.** *Diatom Res* 2004, **19**(2):203-228.
10. Alverson AJ, Kang S-H, Theriot EC: **Cell wall morphology and systematic importance of *Thalassiosira ritscheri* (Hustedt) Hasle, with a description of *Shionodiscus* gen. nov.** *Diatom Res* 2006, **21**(2):251-262.
11. Hustedt F: **Die Diatomeenflora des Küstengebietes der Nordsee vom Dollart bis zur Elbemündung. I. Die Diatomeenflora in den Sedimenten der unteren Ems sowie auf den Watten in der Leybucht, des Memmert und bei der Insel Juist [Diatom flora of the North Sea coastal region from Dollart to the Elbe estuary. I. Diatom flora in sediments of the lower Ems and on the intertidal flats of the Leybucht, the Memmert and at the Island Juist]**. *Abhandlungen des Naturwissenschaftlichen Verein zu Bremen* 1939, **31**(571-677).
12. Alverson AJ, Jansen RK, Theriot EC: **Bridging the Rubicon: Phylogenetic analysis reveals repeated colonizations of marine and fresh waters by thalassiosiroid diatoms**. *Mol Phylogenet Evol* 2007, **45**(1):193-210.
13. Armbrust EV, Berges JA, Bowler C, Green BR, Martinez D, Putnam NH, Zhou S, Allen AE, Apt KE, Bechner M *et al*: **The genome of the diatom *Thalassiosira pseudonana*: Ecology, evolution, and metabolism**. *Science* 2004, **306**(5693):79-86.
