# Supplementary material for: The model marine diatom Thalassiosira pseudonana likely descended from a freshwater ancestor in the genus Cyclotella
Source: BMC Evol Biol. 2011 May 14;11:125. doi: 10.1186/1471-2148-11-125 (PMC3121624; doi:10.1186/1471-2148-11-125)
Supplement: Additional file 5 — Hustedt's observations on the type locality of Cyclotella nana (= T. pseudonana). [file 1471-2148-11-125-S5.PDF]

Additional File 5. Hustedt's observations on the type locality of *Cyclotella nana* (=*Thalassiosira pseudonana*)

Hustedt described *Cyclotella nana* in his 1957 treatment of the tributaries of the River Weser near the city of Bremen [ref. 1, p. 212]. His description is short (Additional file 4) and does not refer to either a type specimen or a sample, but he noted that the species was "widespread and often frequent to plentiful" in the Wümme region, both in planktonic and sediment samples. During typification of Hustedt's taxa, Simonsen [2] did not find any slides labeled *C. nana*, so he designated a slide on which two *C. nana* specimens had been circled by Hustedt as the lectotype. The selected lectotype slide (BRM 380/36) comes from a sediment sample from the River Wümme taken at the crossing of Ritterhuder street (approximately at 53°09'52" N, 8°45'08" E), which corresponds to one of the localities where Hustedt observed *C. nana* in high abundance.

The River Wümme connects to the tide-affected lower part of the River Weser through the short river stretch called Lesum (roughly 10 km long), which arises from the confluence of two rivers, the Wümme and the Hamme. For his 1957 book [1], Hustedt sampled the Lesum and the Wümme between the confluence and the town of Borgfeld (roughly 20 km upstream). The latter is the location of the most downstream dam in the river, which marks the boundary of tidal effects. According to his original notes, Hustedt observed *C. nana* at all these Wümme and Lesum sampling sites, and he noted particularly high abundances in samples at the crossing of Ritterhuder Street (Ritterhuder Heerstrasse) and in the Lesum at Burg (see below). This area is under a weak tidal influence, as also illustrated in Hustedt [1], in which he gave chloride concentrations for this stretch. When converting his chloride concentrations given to NaCl concentrations, they are in the range of 0.5–0.8% salinity for the Wümme and somewhat higher, in the range of 1.1–4.4% for the Lesum.

Hustedt's original notes contain two additional pieces of information about the Wümme and Lesum samples that were not included in the original publication [1]. The typed notes include a listing of species found in each sample, and a table (13 typed-pages in length) lists the distribution of each taxon, integrated across the sampling localities described above. The notes illustrate that at the *C. nana* type locality, Hustedt found *C. nana*, *C. pseudostelligera* (now *Discostella pseudostelligera*), and *C. atomus*. By this time, Hustedt had already described the latter two species himself, indicating that he was able to differentiate these three species under the light microscope.

The following table was extracted from Hustedt's original notes about his diatom samples from the Wümme and Lesum localities, which together constitute the type material for *Cyclotella nana*. The notes summarize the relative abundances of *Cyclotella* (C), *Stephanodiscus* (S), and *Thalassiosira* (T) species in the region. Abundance codes are: ss = very rare ("sehr selten"); + = present; h = frequent ("häufig"); sh = very frequent ("sehr häufig"); m = plentiful ("massenhaft"). Sampling locality codes are: 1 = Borgfeld; 2 = Gehrden; 3 = crossing Ritterhuder Street (*C. nana* lectotype locality); 4 = Villa Marssel; 5 = Burg; 6 = Lesum; 7 = Grohn. We have not shown his notes about an effluent ditch at Villa Marßel, a mansion that since has been demolished.

	1	2	3	4	5	6	7
C. atomus		h	sh	h	h	+	h
C. comta		ss			ss		
C. meneghiniana	+	h	sh	sh	sh	h	h
C. meneghiniana v. laevissima		+				+	
C. nana		+	sh	h	sh	+	h
C. pseudostelligera	+	+	h	+	+	+	+
C. striata	+	sh	m	sh	m	m	m
S. astraea				ss	ss		ss
S. minutula					ss	s	ss
S. dubius		+		+	+	+	+
S. hantzschii	+	+	+	+	h	+	+
S. lucens		+	+		+		+
S. subtilis		+	sh	h	h	+	h
S. tenuis		+	h	+	+	+	+
T. decipiens			+		+	+	+
T. fluviatilis		+	+	h	+	+	+
T. visurgis[1]		+					+

[1]Added in handwriting

References

1. Hustedt F: **Die Diatomeenflora des Flußsystems der Weser im Gebiet der Hansestadt Bremen [Diatom flora of the tributaries of the Weser near the city of Bremen]**. *Abh Naturw Ver Bremen* 1957, **34**:181-440.
2. Simonsen R: **Catalogue of the Diatom Types of Friedrich Hustedt. Volume 1.**, vol. 1. Berlin: J. Cramer; 1987.
